# Unexpected plateauing of childhood obesity rates in developed countries

**DOI:** 10.1186/1741-7015-12-17

**Published:** 2014-01-31

**Authors:** Martin Wabitsch, Anja Moss, Katrin Kromeyer-Hauschild

**Affiliations:** 1 Division of Pediatric Endocrinology and Diabetes, Interdisciplinary Obesity Unit, Department of Pediatrics and Adolescent Medicine, Ulm University, Eythstr. 24, D-89073 Ulm, Germany; 2 Institute of Human Genetics, Jena University Hospital, Friedrich-Schiller-University Jena, Kollegiengasse 10, D-07740 Jena, Germany

**Keywords:** Obesity, Children and adolescents, Extreme obesity, Prevalence, Trend societal and environmental determinants

## Abstract

Surveys performed in the past 10 to 15 years show a yet unexplained stabilization or decline in prevalence rates of childhood obesity in developed countries. The projected continuous increase in obesity prevalence throughout future decades seems not to occur at present. Apparently, saturation has been reached, which might be related to societal adjustments. Hence, we postulate a cumulative effect of public health programs for obesity prevention resulting, for example, in an increase in physical activity, and a decline in television viewing and in the consumption of sugar-sweetened soft drinks by children. Effective public health programs are urgently needed for developing countries, where obesity rates in children still continued to increase during the past decade.

## Introduction

One of the most striking changes in human biology, starting from around 1980, has been the worldwide dramatic increase in prevalence rates of overweight and obesity in children [1]. This impressive development has recently been followed by stabilization or even decline in prevalence rates [2-10]. This commentary highlights the published data on the recent stabilization in childhood obesity rates. These data and their interpretation can serve as a basis for future prevention programs.

### Past and recent trends in childhood obesity

The body mass index (BMI), defined as weight/(height)^2^ is a surrogate parameter for body fat mass in adults and in children [11]. There are only a few datasets describing the cross-sectional development of BMI values in children over several decades before 1980. These data show a rather stable or slowly increasing prevalence of childhood obesity [12-15]. However, between 1980 and 2000, mean BMI values in children and the rates of childhood overweight and obesity increased dramatically in many countries [1]. This was paralleled by a steep increase in skinfold thickness, which is an anthropometric indicator of the amount of subcutaneous fat, and is widely used to assess body fat [16]. Skinfold thickness increased not only in overweight children, but also in normal and underweight children [17].

Along with the increase in obesity prevalence, the BMI distribution shifted in a skewed fashion, indicating that the heaviest children had become even heavier [18]. This matched the observation that the numbers of extremely obese children and adolescents (BMI >99th percentile) increased to a greater degree than those of individuals in other obesity categories (BMI 95th to 96.9th and BMI 97th to 98.9th percentile) [19,20].

Starting at around the year 2000, childhood obesity rates apparently reached a plateaue or even declined in developed countries [2-10]. This was an unexpected finding, because, for example, in the USA, it has been suggested that the prevalence rate of obesity in children will reach 30% by 2030 [21]. Recent data from the National Health and Nutrition Examination Survey (NHANES) now show that the rapid increase in obesity prevalence rates seen in the 1980s and 1990s has not continued [5]. When sex differences were analyzed, the flattening was more marked for girls than for boys. Furthermore, there were age-related differences, with prevalence declining more in preschool children (aged 2 to 5 or 6 years) compared with primary school aged children (6 to 11 or 12 years) or adolescents (12 to 19 years) [6]. However, it should be noted that extreme obesity is still increasing, despite the declining rates for lower obesity categories [19,20].

It is interesting that also in China, a developing country, stabilization of obesity rates has been observed. In the Jiangsu Province, no increase in the rate of overweight or obesity was seen in 12 to 14-year-old students in both urban and rural areas [6]. We have summarized all published data known to us that report a leveling off or a decline in prevalence rates of childhood overweight and obesity in Table 1. However, it should be noted that in all of the reported countries, the prevalence rates are still at a high level and still significantly higher than before 1980.

**Table 1:** Compilation of published data on stabilization or decline in prevalence rates of overweight and obesity in children in different countries

**Author**	**Publication year**	**Country**	**Year**	**Age group, years**
Olds *et al*.	2010	Australia	1985 to 2008	2 to 18
Shi *et al*.	2005	China-Jiangsu Province	2002 to 2007	12 to 14
Ministry of Health	2003; 2008	New Zealand	2002 to 2006/7	5 to 14
Lissner *et al*.	2010	Sweden	1999 to 2005	10 to 11
Murer *et al*.	2013	Switzerland	1999 to 2012	6 to 12
Aeberli *et al*.	2009	Switzerland	2002 to 2007	6 to 13
de Wilde *et al*.	2009	The Hague, Netherlands	1999 to 2007	3 to 16
NHS Information Centre (NCMP)	2010	England	1995 to 2007	2 to 15
Ministère de la Santé	2010	France	1999 to 2007	5 to 15
Salanave *et al*.	2009	France	2000 to 2007	7 to 9
Péneau *et al*.	2009	France	1996 to 2006	6 to 15
Lioret *et al*.	2009	France	1999 to 2007	3 to 14
Ogden *et al*. (NHANES)	2012	USA	1999 to 2010	2 to 19
Moss *et al.*	2012	Germany	1992 to 2009	5 to 7
Blüher *et al*.	2011	Germany	1999 to 2008	4 to 16
Schmidt Morgen *et al*.	2013	Denmark	1998 to 2011	3 mo to 16 yrs
Mitchell *et al*.	2007	Scotland	1997 to 2004	5,66
Tambalis *et al*.	2010	Greece	1997 to 2007	8 to 9
Schnohr *et al*.	2005	Greenland	1980 to 2004	6 to 7
CDC	2013	Anchorage, Alaska	2003/4 to 2010/11	5 to 12
Popkin *et al*.	2006	Russia	1995 to 2004	10 to 17,9

*Abbreviations*: CDC, Centers for Disease Control and Prevention; NHANES, National Health and Nutrition Examination Survey; NHS, National Health Service; NCMP, National Child Measurement Programme.

### Possible causes and consequences of the observed trends in childhood obesity

**Figure 1:**
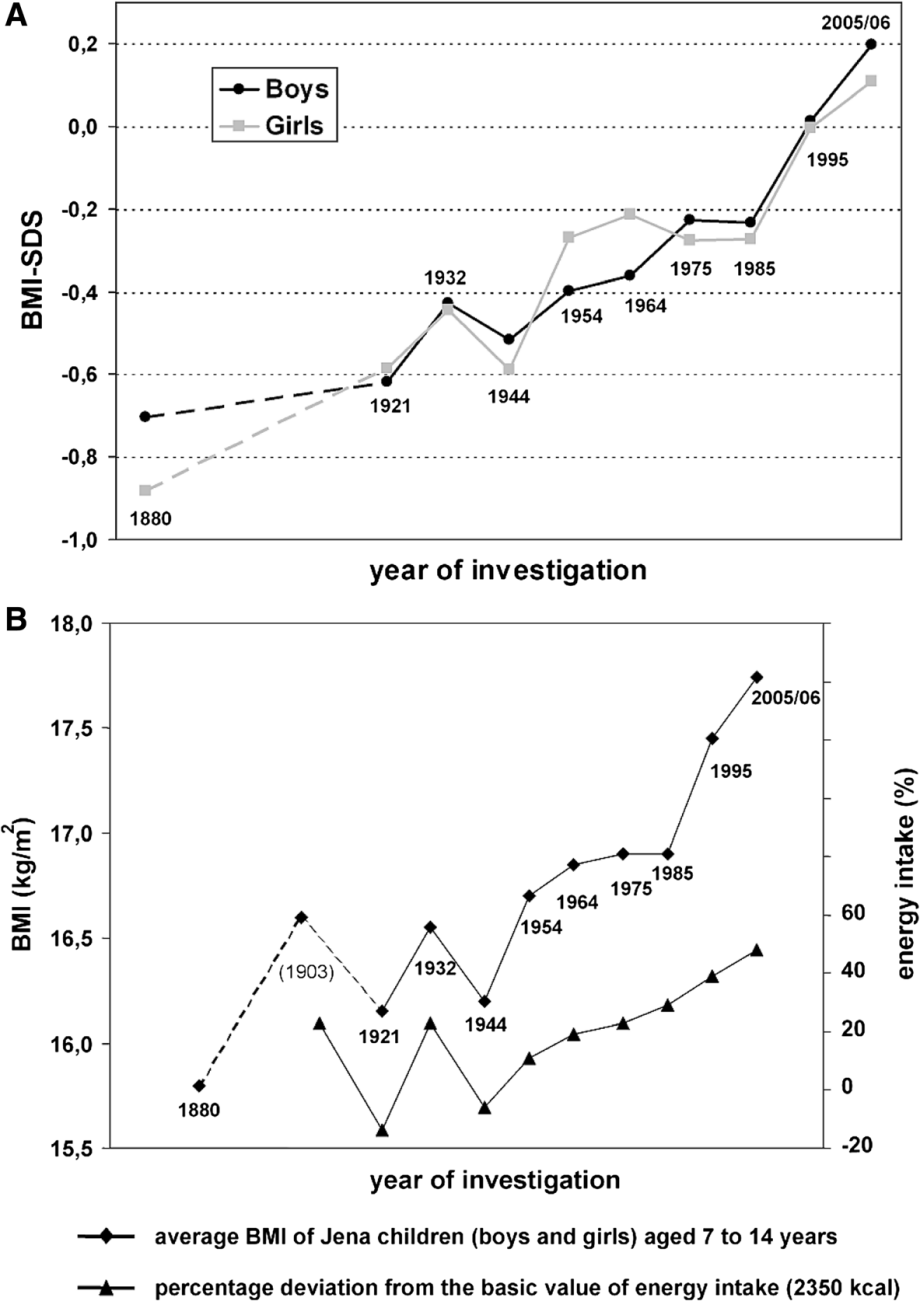
**Changes in BMI parallel changes in living conditions and energy intake over time. (A)** Development of body mass index standard deviation score (BMI SDS) of Jena schoolchildren (7 to 14 years of age) between 1880 and 2005. The children’s average BMI SDS values increased slightly in the time period between 1880 and 2005/06, corresponding to a BMI increase of 1.8 kg/m^2^ (0.14 kg/m^2^ per decade) in boys and of 2.1 kg/m^2^ (0.17 kg/m^2^ per decade) in girls. This increase did not occur continuously. The marked increase in average BMI SDS between 1921 and 1932 indicates nutritional normalization following the famine due to World War I. This was followed by a downward shift in mean BMI between 1932 and 1944, reflecting a deterioration in living conditions during World War II. The marked increase in BMI SDS after 1985 was associated with a substantial increase in prevalence rate of obesity [22], and is a result of the dramatic changes in living conditions due to the German reunification. **(B)**: Development and association of BMI and energy intake of Jena school children (7 to 14 years of age) between 1880 and 2005 (reproduced with permission from Zellner *et al.*[15]). The figure shows that BMI values as a surrogate of body fat mass paralleled the changes in energy intake over a time period of 100 years.

Changes in BMI as a surrogate of body fat mass parallel changes in living conditions and energy intake over time. This has been shown in a study of children in Jena over a period of more than 100 years (Figure 1) [15]. This study used measurements based on 10 anthropological investigations carried out between 1880 and 2005/2006. It belongs to the longest running longitudinal studies of schoolchildren in a single community worldwide, and provides a unique dataset to analyze secular changes in the physical development of schoolchildren.

The dramatic increases in obesity prevalence rates and body fat after 1980 are related to changes in individual behaviors of children. Children nowadays have decreased physical activity, and increased screen time and consumption of energy-dense foods and snacks [1]. These behavioral changes are probably related to social and environmental changes that affect the whole population [18].

In return, the recent decline in obesity prevalence rates in children in developed countries may be the result of a cumulative effect of programs designed to prevent childhood obesity [23-25]. After 1980, the recognition by healthcare professionals, schools, community organizations, industry, and governments of obesity as a health problem increased. Both at national level and at state and local level, programs focusing on reducing the consumption of energy-dense foods and television viewing, as well as increasing the daily physical activity have been developed to reduce environmental factors contributing to inappropriate weight gain [23-25]. Although it is possible that a biological plateau for obesity has been reached, there are several hints indicating that these initiated public health efforts have contributed to the leveling off of obesity rates.

A very recent study showed significant increases in daily physical activities and consumption of fruits and vegetables between 2001 and 2010 in a nationally representative cohort of students in grades 6 to 10. Television viewing and the consumption of sweets and sweetened beverages decreased during the same time period [26]. Another supporting example is the recently observed decline in the consumption of sugar-sweetened soft drinks by children, which parallels the decline in obesity prevalence rates [27,28]. These examples support the notion that in countries facing the childhood obesity epidemic over several years, the initiated public health programs have been able to stop the increasing obesity trends by influencing the lifestyle of children. If this can be confirmed in further analyses, such programs might even be improved in order to achieve a yet better success rate.

However, in contrast to these findings in developed countries, recent prevalence rates of overweight and obesity in children in developing low and middle income countries are still increasing at large [29]. This seems to be because the western lifestyle with easily available and low cost energy-dense food and increased motorization started to develop later in these countries, and had now become increasingly adopted [29]. These countries may be able to adapt public health programs from developed countries to their situation and launch them immediately in order to prevent further increases in childhood overweight and obesity rates, and possibly reach a plateau at a lower level than seen in developed countries.

## Conclusions

The BMI of children is sensitive to living conditions and lifestyles, and the deep changes in children’s living conditions and lifestyles in modern societies resulted in an extraordinary increase in childhood obesity rates from the 1980s. Unexpectedly, a plateau or even a decline in prevalence rates has been reported for several developed countries during the past 10 to 15 years. There are hints indicating that public health programs in these countries aiming at reducing obesity-promoting lifestyles might be responsible for the leveling off in obesity rates. However, it has to be recognized that despite the reported stabilization the prevalence rates of overweight and obesity in children, these rates remain at a high level and still represent a significant health issue.

## Competing interests

The authors declare that they have no competing interests.

## Authors’ contributions

MW performed the literature research, wrote the first draft of the manuscript, and completed the final version. AM wrote sections of the manuscript, created figures and formatted the manuscript. KKH wrote sections of the manuscript and created figures. All authors read and approved the final manuscript.
